# Kindlin-1 Regulates Integrin Dynamics and Adhesion Turnover

**DOI:** 10.1371/journal.pone.0065341

**Published:** 2013-06-11

**Authors:** Coert Margadant, Maaike Kreft, Giovanna Zambruno, Arnoud Sonnenberg

**Affiliations:** 1 Division of Cell Biology, The Netherlands Cancer Institute, Amsterdam, The Netherlands; 2 Laboratory of Molecular and Cell Biology, IDI-IRCCS, Rome, Italy; NCMLS, Radboud University Nijmegen Medical Center, The Netherlands

## Abstract

Loss-of-function mutations in the gene encoding the integrin co-activator kindlin-1 cause Kindler syndrome. We report a novel kindlin-1-deficient keratinocyte cell line derived from a Kindler syndrome patient. Despite the expression of kindlin-2, the patient’s cells display several hallmarks related to reduced function of β1 integrins, including abnormal cell morphology, cell adhesion, cell spreading, focal adhesion assembly, and cell migration. Defective cell adhesion was aggravated by kindlin-2 depletion, indicating that kindlin-2 can compensate to a certain extent for the loss of kindlin-1. Intriguingly, β1 at the cell-surface was aberrantly glycosylated in the patient’s cells, and its expression was considerably reduced, both in cells *in vitro* and in the patient’s epidermis. Reconstitution with wild-type kindlin-1 but not with a β1-binding defective mutant restored the aberrant β1 expression and glycosylation, and normalized cell morphology, adhesion, spreading, and migration. Furthermore, the expression of wild-type kindlin-1, but not of the integrin-binding-defective mutant, increased the stability of integrin-mediated cell-matrix adhesions and enhanced the redistribution of internalized integrins to the cell surface. Thus, these data uncover a role for kindlin-1 in the regulation of integrin trafficking and adhesion turnover.

## Introduction

Integrins are αβ heterodimeric transmembrane glycoproteins that link the extracellular matrix to the cytoskeleton. Integrin-ligand binding triggers the recruitment of a variety of adaptor, structural, and signalling proteins, and the formation of adhesion complexes such as focal adhesions (FAs) [1], [2]. Cell adhesion to the extracellular matrix is crucial for the integrity of tissues, in particular for those that encounter great mechanical stress. In the skin, integrins provide for the attachment of the epidermis to the underlying basement membrane (BM). The main epidermal integrin is the laminin (Ln)-binding integrin α6β4, which is localized in hemidesmosomes and connects to intermediate filaments [3]. In addition, β1-integrins such as the collagen (Col)-binding α2β1, Ln-binding α3β1, and the RGD-binding α9β1 integrins, which connect to the actin cytoskeleton, are expressed in basal keratinocytes [4], [5].

Many integrins can tune their affinity for ligand by conformational changes, and the switch from the low- to the high-affinity conformation is called integrin activation [6]. Integrin activation is promoted by the binding of talin-1 or talin-2 and any of the 3 kindlin isoforms to the cytoplasmic tail of the β-subunit [6]–[8]. The kindlins consist of an F0–F3 four-point-one/ezrin/radixin/moesin (FERM) domain, that contains the integrin-binding site in F3, and a pleckstrin homology (PH) domain inserted into F2. Kindlin-1 is expressed at high levels in epithelia, in particular in the epidermis and the gastro-intestinal tract, and loss-of-function mutations in *KIND1*, the gene encoding kindlin-1, cause Kindler syndrome (KS), a congenital bullous disorder of the epidermolysis bullosa-type [9]–[11].

KS is characterized by skin fragility and blistering, photosensitivity and poikiloderma, while some patients also suffer from colitis [12]–[17]. Hemidesmosomes appear unaffected in KS patients and the defects result from compromised function of β1-integrins. Indeed, the defects are reminiscent of the abnormalities in mice lacking the α3 or the β1 subunit in the epidermis, as well as of patients carrying mutations in the *ITGA3* gene encoding α3 [12]–[22]. *In vitro*, keratinocytes isolated from KS patients or keratinocytes in which kindlin-1 expression is suppressed, display several abnormalities related to defects in β1 integrin function, including reduced cell adhesion, cell spreading, and polarity [23]–[25].

In this paper, we describe a novel kindlin-1-deficient keratinocyte cell line derived from an Italian KS patient, which expresses kindlin-2 but not kindlin-1. We investigated functional redundancy between the kindlins, and identified a role for kindlin-1 in the regulation of adhesion turnover and integrin trafficking.

## Results and Discussion

### Defects in β1 Integrin Function in Kindler Syndrome Cells that Express Kindlin-2 but not Kindlin-1

**Figure 1:**
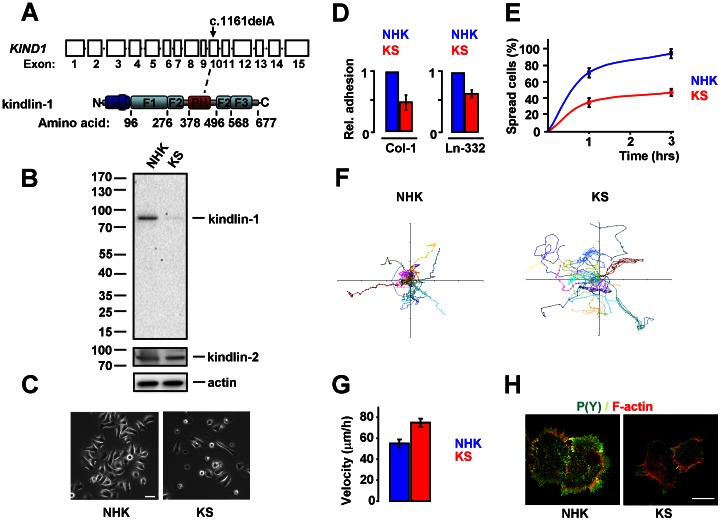
Abnormalities in KS cells. **A**) Schematic representation of the *KIND1* gene (top), indicating the position of the c.1161delA mutation, and kindlin-1 protein (bottom). Exons are represented by boxes, introns are not to scale. **B**) Western blot showing the expression of kindlin-1 and kindlin-2 in NHK and KS cells. **C**) Phase/contrast images of NHK and KS cells. Bar, 20 µm. **D**) Adhesion of KS cells to Col-1 and Ln-332, expressed relative to that of NHK. Shown are the averages ±SEM from 3 independent experiments. **E**) Cell spreading of NHK and KS cells on Col-1. Shown are the averages ±SEM from 3 independent experiments. **F**) Rose-plots depicting migration tracks of NHK and KS cells. **G**) Quantification of the velocity of cell migration (Bars represent averages ±SEM from ∼250 cells out of 3 experiments). **H**) Confocal images of FAs, visualized using an antibody against P(Y) (green), and F-actin (red). Scale bar, 10 µm.

We isolated kindlin-1-deficient keratinocytes from a previously described male KS patient from Italy [26]. This patient is homozygous for the mutation c.1161delA within exon 10 (Fig. 1A). We first investigated kindlin-1 protein expression by Western blotting, using an antibody directed against an epitope in the F1 domain [24]. Full-length kindlin-1 was clearly detected at the expected size (∼75 kDa) in lysates of normal human keratinocytes (NHK) isolated from a healthy individual [27], but not KS cells (Fig. 1B). In contrast, kindlin-2 expression was detected in both NHK and KS keratinocytes (Fig. 1B). The morphology of the KS cells was highly abnormal, as compared to that of NHK (Fig. 1C). In addition, cell growth was compromised and large numbers of dead cells were regularly observed in KS, but not in NHK cultures (Fig. 1C and unpublished data). To establish whether the observed abnormalities were due to defects in integrin-mediated adhesion, we measured cell adhesion to Col-1, a ligand for integrin α2β1, and to a Rac-11P cell-derived matrix rich in Ln-332, a ligand for integrin α3β1 [21]. Adhesion of KS cells to these ligands was indeed significantly impaired, and only a fraction of the adherent cells spread properly over the substratum (Figs. 1D,E). Consistent with reduced integrin-mediated adhesion, cell motility was significantly enhanced (Figs. 1F,G). We and other groups have previously observed a similar increase in keratinocyte migration upon loss of α3β1, both in cultured keratinocytes and in the epidermis [21], [28]–[30]. However, it should be noted that conflicting results have been reported with regard to keratinocyte migration in the absence of kindlin-1 [24], [31]. Similarly, either impaired or increased migration have been described for keratinocytes isolated from epidermolysis bullosa patients that lack Ln-332 [32]–[36]. Discrepancies in cell migration between different cell lines may derive from the integrin repertoire expressed on the cell-surface, the relative abundance of a particular integrin, and the density of available ligand. Indeed, as migrating cells must be able to attach and exert traction, but also to detach from the substratum, the velocity of migration is a biphasic function of adhesion strength [37]. Finally, we studied the organization of the actin cytoskeleton and the presence of FAs, using phalloidin and an antibody against phosphotyrosines P(Y). P(Y)-staining was generally weak in KS keratinocytes, and actin filaments appeared abnormal (Figure 1H). In contrast, the synthesis and deposition of Ln-332 in KS cells was not impaired, although the pattern of deposition seemed different from that of NHK cells (Fig. S1). However, because patterns of Ln-332 deposition can differ considerably between cell lines, which often reflects differences in cell motility [21], [29], [38], it is uncertain whether the differences observed here are a direct consequence of kindlin-1 loss. In summary, we have isolated a kindlin-1-deficient keratinocyte cell line that displays defects in β1 integrin function, despite the presence of kindlin-2.

### Kindlin-1 and Kindlin-2 are Partially Redundant, and β1 Expression is Decreased in Kindlin-1-deficient Keratinocytes and Epidermis

**Figure 2:**
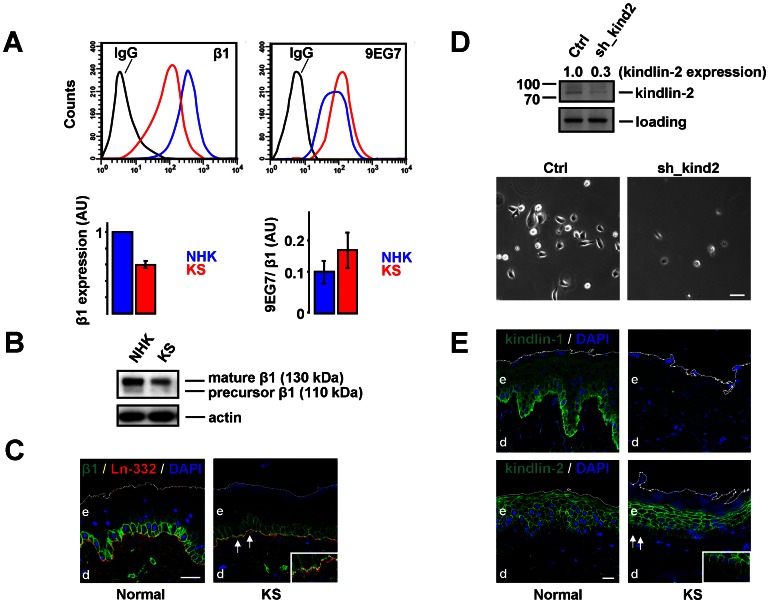
Decreased integrin expression in the absence of kindlin-1. **A**) FACS histograms (top) and quantification (bottom; average ±SEM from 3 independent experiments) of NHK and KS cells showing cell-surface expression of β1 (left) and active β1 (right), as measured by 9EG7 staining. AU, arbitrary units. **B**) Western blot showing the precursor β1 (110 kDa) and the mature form of β1 (130 kDa) in NHK and KS cells. **C**) Expression of β1 (green) and Ln-332 (red) in the skin of an unaffected individual (normal) and the KS patient. The upper border of the epidermis is indicated with a white line. Bar, 50 µm. d; dermis, e; epidermis. **D**) Depletion of kindlin-2 causes detachment of KS cells. The numbers above the blot indicate the normalized kindlin-2 expression in the remaining (attached) cells, relative to that in untreated cells. Bar, 20 µm. **E**) Expression of kindlin-1 (top) and kindlin-2 (bottom) in the skin. Bar, 50 µm. d; dermis, e; epidermis.

We next investigated the cell-surface expression and activation status of β1-integrins in KS and NHK cells by flow cytometry. Interestingly, β1 cell-surface expression was significantly reduced (about two-fold), whereas the activation status, as judged by the ratio of 9EG7 staining over total β1 staining, was slightly (but not significantly) increased (Fig. 2A). The reduction of β1 levels at the cell surface was accompanied by reduced expression of associated α2 and α3-subunits, while β4 levels were normal (Fig. S2). Decreased expression of β1 in KS cells was further confirmed by Western blotting (Fig. 2B). We then analysed skin biopsies of the same patient. Ln-332 staining revealed BM abnormalities and detachment of keratinocytes in the patient’s epidermis (Fig. 2C; indicated by arrows), typical of KS [24], [39], and the expression of β1 was strikingly decreased (Fig. 2C). Thus, whereas there is a clear reduction in β1 expression, both *in vivo* and *in vitro*, β1 activation in the KS cells is not impaired. The latter finding is reminiscent of keratinocytes derived from the kindlin-1 knockout mice, in which integrin-mediated cell adhesion and cell spreading were compromised whereas there was no significant reduction in integrin activation, due to the presence of kindlin-2 [40]. We therefore introduced shRNAs directed against kindlin-2 into KS cells by lentiviral transduction. Depletion of kindlin-2 caused massive detachment of KS cells (Fig. 2D). Previous studies have reported both overlapping and distinct functions of kindlin-1 and kindlin-2 in keratinocytes [41], [42]. Our results are in line with these findings as kindlin-2 can apparently partially rescue cell adhesion in the absence of kindlin-1, but considerable defects in cell adhesion and spreading remain. *In vivo*, kindlin-2 cannot completely compensate for the loss of kindlin-1, either in the epidermis of KS patients, or in the colon of kindlin-1(−/−) mice [40], [43], which is probably due to differences in subcellular localization [11]. We therefore investigated kindlin-2 distribution *in vivo*. Consistent with its expression in NHK and KS cells, kindlin-2 was detected both in the patient’s epidermis and in the epidermis of a normal individual. In basal keratinocytes kindlin-2 localization was exclusively lateral, while kindlin-1 distribution in normal epidermis was predominantly basal, in line with previous observations (Fig. 2E) [11], [43]. Interestingly, kindlin-2 staining at the lateral membranes was weak and occasionally completely absent from the basal keratinocyte layer of the patient (Fig. 2E; indicated by arrows), which most likely reflects defects in cell-cell contacts as we described previously [44].

Together, these data show that β1 expression is reduced in KS cells and epidermis, and that kindlin-2 compensates only partially for reduced cell adhesion in the absence of kindlin-1.

### Stable Re-expression of Kindlin-1 in KS Cells Restores the Defects in Integrin Function

**Figure 3:**
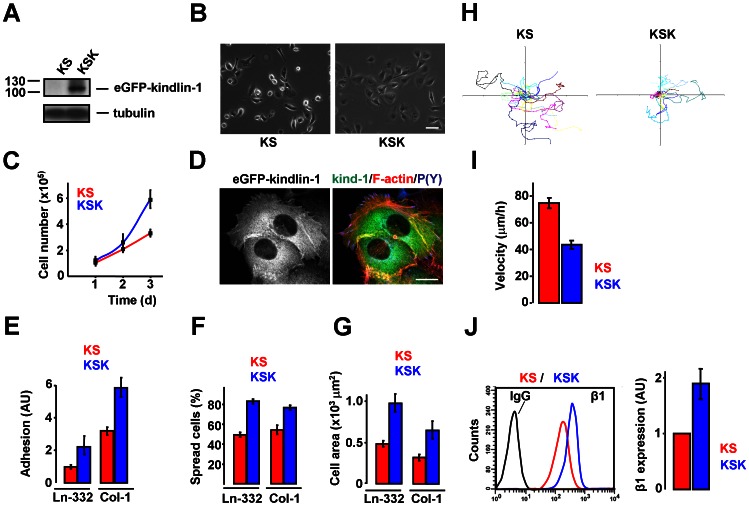
Re-expression of kindlin-1 in KS cells. **A**) Western blot showing expression of eGFP-kindlin-1 in KS and KSK cells. **B**) Morphology of KS and KSK cells. Bar, 20 µm. **C**) Proliferation of KS and KSK cells. Shown are the averages ±SEM from 3 independent experiments. **D**) eGFP-kindlin-1 (green), FAs (blue) and F-actin (red) in KSK cells. Bar, 5 µm. **E**) Cell adhesion to Col-1 and Ln-332 in KS and KSK cells. Bars represent averages ±SEM of 3 independent experiments. AU, arbitrary units. **F**) Number of KS and KSK cells spread on Ln-332 and Col-1. Shown are the average values ±SEM from ∼500 cells out of a representative experiment. **G**) Surface area of KS and KSK cells on Ln-332 and Col-1. Shown are the averages ±SEM from ∼500 cells of a representative experiment. **H**) Rose-plots depicting migration tracks of KS and KSK cells generated by time-lapse video microscopy. **I**) Quantification of the velocity of cell migration (average ±SEM from ∼300 cells out of 3 experiments). **J**) FACS histograms of NHK and KS cells showing β1 cell-surface expression (left) and quantification (average ±SEM from 3 independent experiments) (right). AU, arbitrary units.

The previous sections have shown that in the absence of kindlin-1, integrin-dependent events are disturbed despite the presence of kindlin-2. To investigate whether the observed defects in KS cells are a direct consequence of the loss of kindlin-1, kindlin-1 expression was restored in KS cells by retroviral delivery of eGFP-conjugated kindlin-1 followed by FACS sorting, creating a stable cell line that we designated KSK. Expression of eGFP-kindlin-1 was confirmed by Western blotting (Fig. 3A). Kindlin-1 restored the aberrant morphology of KS cells to normal keratinocyte morphology (Fig. 3B, compare to Fig. 1C), and significantly enhanced cell proliferation (Fig. 3C). Kindlin-1 was diffusely distributed in the cytoplasm, while some enrichment in P(Y)-positive FAs was observed. In addition, a re-organization of the actin cytoskeleton into stress fibers and/or circumferential actin bundles was observed in KSK cells (Fig. 3D). Re-expression of kindlin-1 reversed the two-fold decrease in cell adhesion as compared to NHK cells, both to Col-1 and to Ln-332-containing matrix (Figs. 3E, 1D). Moreover, the decrease in the number of spread cells was similarly reversed (Figs. 3F, 1E), and the average surface area of KSK cells was about two-fold greater than that of KS cells (Figure 3G). Finally, the enhanced migration of KS cells was decreased by re-introduction of kindlin-1 (Figs. 3H,I and Figs. 1F,G). To investigate whether the re-expression of kindlin-1 also normalized integrin expression on the cell-surface, we analyzed β1 cell-surface levels by flow cytometry. Expression of β1 on the plasma membrane was up to two-fold higher in KSK cells than in KS (comparable to those in NHK) (Figs. 3J, 2A). The promoting effect of kindlin-1 on integrin cell-surface levels is in line with several previous studies [45]–[47], and was further supported by the observation that overexpression of kindlin-1 also enhanced β1 cell-surface expression in NHK cells, which was accompanied by increased cell spreading (Fig. S3).

Together, these data show that kindlin-1 expression in KS cells rescues the defects in β1 integrin function, and restores the KS phenotype to that of normal keratinocytes.

### Regulation of β1 Expression and Function by Kindlin-1 Requires the F3 Domain

**Figure 4:**
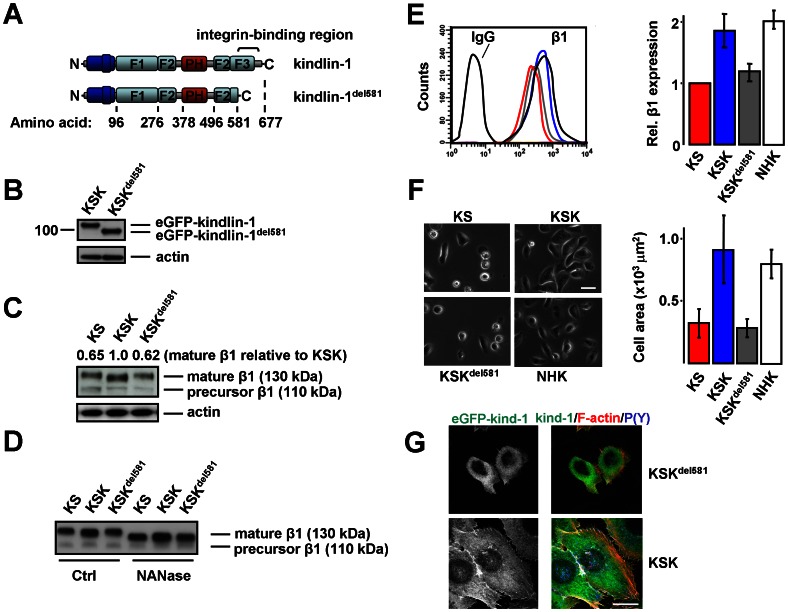
Regulation of β1 expression and cell spreading by kindlin-1 require the F3 domain. **A**) Schematic representation of full-length kindlin-1 (top) and kindlin-1^del581^ (bottom). **B**) Expression of full-length eGFP-kindlin-1 and eGFP-kindlin-1^del581^ in KSK and KSK^del581^ cells. **C**) Expression of precursor β1 (110 kDa) and mature β1 (130 kDa) in KS, KSK, and KSK^del581^ cells. Expression of mature β1 was quantified by densitometry, normalized to actin, and expressed relative to the expression in KSK cells. Shown are the values acquired from a representative blot. **D**) Immunoprecipitated β1 was treated with neuraminidase (NANase) and analyzed by Western blotting. **E**) FACS histograms (left) and averages ±SEM quantified from 3 independent experiments (right) of β1 cell-surface expression in KS, KSK, KSK^del581^, and NHK, expressed relative to that in KS. **F**) Phase-contrast images of KS, KSK, KSK^del581^ and NHK on Col-1 (left), and average surface area ±SEM of KS, KSK, KSK^del581^ and NHK cells (quantified from ∼250 cells from a representative experiment) (right). Bar, 10 µm. **G**) Subcellular distribution of eGFP-kindlin-1 and eGFP-kindlin-1^del581^ (green). FAs (blue), F-actin (red). Bar, 5 µm.

We next investigated whether the effects of kindlin-1 depend on a direct interaction with the integrin β1-tail. The integrin-binding site in kindlin-1 resides in the C-terminal F3 domain, and a mutation that causes the expression of a protein lacking the F3 and part of the F2 has been identified in a KS patient, demonstrating the vital importance of this region [48]. In addition, we have recently isolated a zebrafish mutant with KS-like epidermal defects, which expresses a truncated kindlin-1 protein lacking the F3 domain [44]. To delete the integrin-binding site, we truncated the F3 region after residue 581, and stably expressed eGFP-kindlin-1^del581^ into KS cells, creating a cell line designated KSK^del581^ (Fig. 4A). Western blotting revealed a band of the expected size of ∼95 kDa in KSK^del581^ cells (Fig. 4B). Intriguingly, increased expression of mature β1, but not precursor β1, was observed in KSK cells but not in KSK^del581^, suggesting that a direct interaction is required for the stimulation of β1 cell-surface expression by kindlin-1. Furthermore, the mobility of mature β1 in gel electrophoresis was reduced in lysates of KS and KSK^del581^ as compared to that of KSK cells (Fig. 4C). This phenomenon was abolished by treatment of immuno-precipitated β1 with neuraminidase, indicating that kindlin-1 regulates β1 sialylation in a manner dependent on the F3 domain (Fig. 4D). Flow cytometry analysis confirmed that whereas the full-length kindlin-1 increased β1 cell-surface expression to wild-type levels (i.e. as in NHK), mutant kindlin-1 did not (Fig. 4E). Furthermore, expression of full-length kindlin-1 enhanced cell spreading about two-fold (comparable to that of NHK), but eGFP-kindlin-1^del581^ did not promote cell spreading (Fig. 4F). Consistently, FAs seemed less pronounced in KSK^del581^ than in KSK cells, and the subcellular distribution of mutant kindlin-1 was different from that of full-length kindlin-1; kindlin-1^del581^ localization seemed predominantly cytoplasmic, with no clear enrichment in adhesions (Fig. 4G). These results are in line with the observation that wild-type kindlin-1, when overexpressed in fibroblasts, is targeted to FAs and increases cell-surface expression of α5β1, whereas kindlin-1 mutants that do not interact with the β1-tail are unable to do so [45].

Together, these data suggest that a direct interaction between kindlin-1 and β1 is required for the targeting of kindlin-1 to cell-matrix adhesions, and for the effects of kindlin-1 on β1 cell-surface expression and glycosylation.

### Targeting of Kindlin-1 to Adhesions and Adhesion Dynamics Depend on the F3 Domain

**Figure 5:**
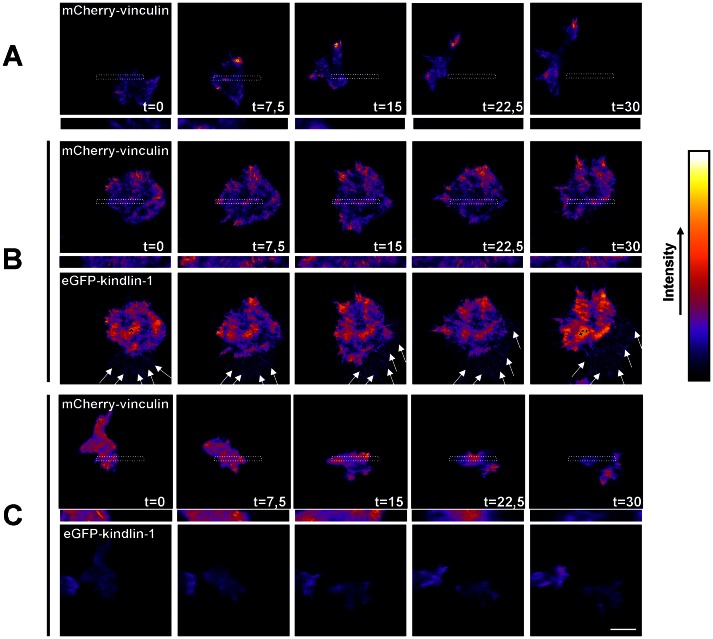
Kindlin-1 targeting to adhesions and adhesion stability depend on the F3 domain. **A**) Stills from a TIRF movie, showing the dynamics of mCherry-vinculin in KS cells. **B**) Dynamics of mCherry-vinculin (top), and eGFP-kindlin-1 (bottom) in KSK cells. **C**) Dynamics of mCherry-vinculin (top), and eGFP-kindlin-1 (bottom) in KSK^del581^ cells. Look-up table ‘fire’ was used to enhance visibility of adhesions. Shown are images at 0, 7.5, 15, 22.5, and 30 min. Boxed regions are enlarged. Arrows indicate retraction fibers. Bar, 10 µm.

We next addressed the relationship between kindlin-1 targeting to adhesions and adhesion dynamics in living cells. To this end, we introduced the FA marker vinculin, fused to mCherry, into KS, KSK, and KSK^del581^ cells by lentiviral transduction. The dynamics of eGFP-kindlin-1 and mCherry-vinculin were then monitored by total internal reflection (TIRF) microscopy. Consistent with the results of the cell migration assays, KS cells were very motile and displayed many rapid shape changes. Imaging of vinculin revealed few FAs that had a high turnover rate (Movie S1 and Fig. 5A). In contrast, KSK cells were considerably more static, in line with the reduced migration speed, and their adhesions were much more stable than those in KS cells (Movie S2 and Fig. 5B). Interestingly, eGFP-kindlin-1 was clearly enriched in adhesions, some of which were surprisingly large, but many of these clusters did not contain mCherry-vinculin, suggesting that kindlin-1 and vinculin can reside in distinct pools of adhesions (Movie S2 and Fig. 5B). Furthermore, kindlin-1 was strongly concentrated in retraction fibers, consistent with the role of kindlin-1 in delaying cell migration. In KSK^del581^ cells, we also observed a rapid turnover of mCherry-vinculin-containing adhesions, as well as fast shape changes. Consistent with the images acquired by confocal microscopy, there was some diffuse localization of eGFP-kindlin-1^del581^ at the basal cell-surface, but clearly no enrichment in adhesions or retraction fibers (Movie S3 and Fig. 5C).

Thus, kindlin-1 controls the dynamics of integrin-mediated cell-matrix adhesions, which is dependent on an intact F3 region.

### Kindlin-1 Interaction with β1 Regulates Integrin Traffic

**Figure 6:**
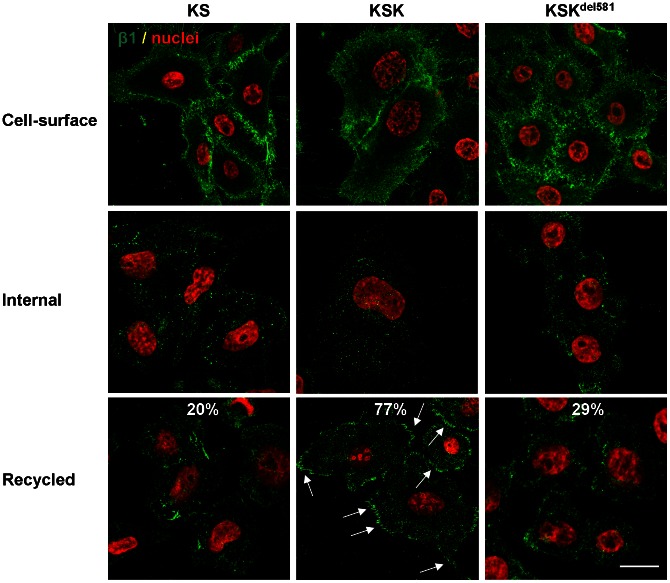
Kindlin-1 interaction with β1 regulates integrin trafficking. Cell-surface β1 integrins on KS, KSK, and KSK^del581^ cells were labelled with DyLight 649-conjugated K-20 at 4°C (top panel), after which they were allowed to internalize in serum-free medium at 37°C for 2 hrs (middle panel). Recycling of the internal pool was induced with 20% FCS for 7.5 min (bottom panel). Cells were then fixed and processed for confocal microscopy. β1 is pseudo-colored green, nuclei were counterstained with DAPI (pseudocolored red). Arrows indicate delivery of recycled β1 to adhesions. Percentages of cells with recycled integrins are shown (from ∼120 cells out of 3 independent experiments). Bar, 10 µm.

We next investigated whether integrin trafficking plays a role in the regulation of cell-matrix adhesion dynamics and β1 surface expression by kindlin-1. Integrins undergo continuous internalization, and the recycling of internalized integrins is important for integrin-mediated processes such as cell spreading [49], [50]. Internalization and recycling were determined according to a well-established protocol [51]. First, we labeled cell-surface β1 with the antibody K20, conjugated to DyLight 649 (10 µg/ml), which clearly revealed localisation of β1 integrins at the membrane in KS, KSK, and KS cells (Fig. 6, top). The cells were then transferred to serum-free medium at 37°C, which allows internalization but not recycling of internalized integrins. The labeled cell-surface pool underwent internalization in all cell lines, with no apparent differences that can be ascribed to kindlin-1 (Fig. 6, middle panel). Recycling of internalized integrins was subsequently induced by stimulation with 20% FCS, which in the majority of KSK cells (77%) triggered the rapid return of β1 integrins to the plasma membrane and their delivery to peripheral adhesions (Fig. 6, bottom). In contrast, redistribution of the internal integrin pool to the plasma membrane was observed only in a small fraction of KS cells (20%) or KS cells expressing truncated kindlin-1 (29%), indicating that kindlin-1 regulates the redistribution of internalized integrins, which is dependent on the F3 domain. A role for kindlin-2 and kindlin-3 in integrin trafficking has been suggested in previous studies, but the mechanism remains to be elucidated [47], [52]. We did not detect kindlin-1 in vesicles, in line with similar observations for kindlin-2 [46], [53]. Therefore, kindlin-1 probably regulates integrin routing indirectly, i.e. by sorting integrins at the plasma membrane to a specific internalization and recycling pathway. This is conceivable as the kindlin-binding site in β1 is largely defined by the membrane-distal NPxY motif, which is also a canonical signal for clathrin-mediated endocytosis.

In summary, the results presented here suggest that kindlin-1 regulates the redistribution of internalized integrins, which requires a direct kindlin-integrin interaction.

## Materials and Methods

### Antibodies, Plasmids and other Materials

Plasmids encoding eGFP-kindlin-1 or mCherry-Vinculin were generously donated by Dr. Reinhard Fassler and Dr. Johan de Rooij, respectively. Antibodies used in this study were directed against actin (clone C4; Chemicon), α-tubulin (clone B5-1-2; Sigma-Aldrich), GFP (Covance), the integrin α6-subunit (GoH3), the integrin β1-subunit (clone TS2/16; Developmental Studies Hybridoma Bank, clone 9EG7; a kind gift from Dr. Dietmar Vestweber, clone K-20; a kind gift from Dr. Andre van Agthoven; and clone 18 from BD Transduction laboratories), the integrin α2-subunit (10G11), the integrin α3-subunit (J143), the integrin α5-subunit (SAM-1), the integrin β4-subunit (439-9B), kindlin-1 (KS-4; a kind gift from Dr. Cristina Has), kindlin-2 (Sigma-Aldrich), kindlin-2 (a kind gift from Dr. Reinhard Fassler), Ln-332 (a kind gift from Dr. Takako Sasaki), plectin (a kind gift from Dr. Katsushi Owaribe), and P(Y) (clone 4G10; a kind gift from Dr. Kevin Wilhelmsen). Neuraminidase, puromycin and zeocin were from Sigma-Aldrich. TRITC-, FITC-, and Cy5-conjugated secondary antibodies, phalloidin, and DAPI were purchased from Molecular Probes (Eugene, OR), HRP-conjugated secondary antibodies were from Amersham, and Col-I was from Vitrogen (Nutacon, Leimuiden, The Netherlands). K-20 was conjugated to DyLight 649 (Thermo Scientific) at the NKI.

### Patient Material, Cell Culture, Cloning, Retroviral and Lentiviral Transductions

The use of skin biopsies and keratinocytes from KS patients for research purposes has been approved by the Local Ethics Committee of the Istituto Dermopatico dell’Immacolata (03/07/2007; study: “Epithelial adhesion disorders: molecular mechanisms, development and validation of diagnostic procedures”). Skin biopsies and primary KS keratinocytes were obtained after written informed consent for use in research from a previously described patient [26], and immortalized by SV40 infection. NHK cells were isolated from human foreskin of a healthy individual, and immortalized by transfection with full-length HPV 16 DNA as described previously [27]. NHK and KS cells were routinely cultured on Col-1 (3 µg/ml) in standard keratinocyte medium (Gibco BRL), supplemented with 50 µg/ml bovine pituitary extract, 5 ng/ml EGF, 100 U/ml penicillin and 100 U/ml streptomycin. Rac-11P cells were cultured in DMEM supplemented with 10% FCS, 100 U/ml penicillin and 100 U/ml streptomycin. All cells were maintained at 37°C and 5% CO_2_. eGFP-kindlin-1^del581^ was generated using eGFP-kindlin-1 in C1. Full-length or truncated kindlin-1 were recloned into LZRS-IRES-zeo, and transfected into Phoenix packaging cells using the Calcium Phosphate method. Virus-containing supernatant was isolated 48 hrs later and stable expression in KS cells was achieved by retroviral transduction, followed by selection with zeocin and cell sorting. Expression of mCherry-vinculin was established by lentiviral transduction of the pLV-CMV-mCherry-Vinculin-Ires-Puro-construct, followed by selection with 5 µg/ml puromycin.

### Knockdown of Kindlin-2 in KS Cells

Short hairpins against human kindlin-2 (target sequence CGACTGATATAACTCCTGAAT), cloned into pLKO.1, were obtained from the TRC shRNA Open Biosystems library and transfected into HEK 293 FT cells together with the Virapower™ Packaging mix (Invitrogen), using Lipofectamine 2000 according to the manufacturers’ instructions. Viral supernatant was harvested 48 hrs later, transduced into KS cells, and positive cells were selected with puromycin.

### Flow Cytometry and Cell Sorting

For flow cytometry and cell sorting, cultured cells were trypsinized, washed twice in PBS containing 2% FCS, and incubated with primary antibodies for 45 min at 4°C. Cells were then washed twice in 2% FCS/PBS, incubated with appropriate secondary antibodies for 45 min at 4°C, washed twice in 2% FCS/PBS, and analyzed on a FACS Calibur (BD Biosciences). Alternatively, the cells were sorted on a MoFlo High Speed Cell Sorter (Beckman Coulter).

### Immunoprecipitations and Western Blotting

Cells were washed in ice-cold PBS and lysed on ice in RIPA buffer (25 mM Tris/HCl pH 7.6, 150 mM NaCl, 1% NP-40, 0.5% sodium deoxycholate, 0.1% SDS), supplemented with protease inhibitor cocktail (Sigma). Immunoprecipitation of β1 was performed essentially as described earlier [21], using TS2/16. For Western blotting of whole-cell extracts, cell lysates were cleared by centrifugation at 13,000×*g*, heated at 95°C in SDS sample buffer (50 mM Tris-HCl pH 6.8, 2% SDS, 10% glycerol, 1% β-mercaptoethanol, 12.5 mM EDTA, 0.02% bromophenol blue), and proteins were resolved by SDS-PAGE, after which they were transferred to polyvinylidene difluoride membranes (Millipore) and analyzed by Western blotting followed by ECL using the SuperSignal system (Pierce Chemical Co.).

### Microscopy

Phase-contrast images were acquired on a Zeiss microscope (Axiovert 25) at 10× (NA 0.25) or 20× (NA 0.3) magnification, using a Zeiss CCD camera (Axiocam MRC) and Zeiss Mr. Grab 1.0 software. For confocal microscopy, cryosections of human skin or cells cultured on coverslips were prepared as previously described [21], and images were acquired on an inverted confocal microscope (Leica AOBS) using 20× (NA 0.7) dry, 40× (NA 1.25) oil, and 63× (NA 1.32) oil objectives (Leica). For TIRF microscopy, cells were seeded on glass coverslips and videos were acquired using Leica application suite software on a Leica DMI600B system with a 63× objective (NA 1.47), at 37°C in an atmosphere containing 5% CO_2_. Images and videos were processed using Photoshop 7.0 and ImageJ 1.44.

### Adhesion, Migration, Cell Spreading, and Proliferation Assays

For adhesion assays, 96-well plates were coated with 2% BSA or 3 µg/ml Col-1 for 1 hr at 37°C. Ln-332-containing matrix was prepared by growing Rac-11P cells to confluency, prior to overnight detachment with 10 mM EDTA at 4°C. The plates were then washed twice with PBS, blocked with 2% BSA for 1 hr at 37°C, and washed twice with PBS before use. Subconfluent cells were trypsinized and seeded at a density of 3×10^4^ cells per well. After 30 min at 37°C, nonadherent cells were removed by washing with PBS. The adherent cells were fixed in 4% PFA, washed with H2O, stained for 10 min with crystal violet, washed with H2O, and then lysed in 2% SDS. Absorbance was measured at 490 nm on a microplate reader. Background values (binding to BSA) were subtracted from all other values.

To determine cell spreading, cells were seeded in 12-well plates coated with Col-1 or Rac-11P matrix. Cells were photographed on a Widefield CCD system using 10× and 20× dry lens objectives (Carl Zeiss MicroImaging, Inc.). The number of spread cells was counted and expressed as a percentage of the total number of cells. Alternatively, the surface area was determined using ImageJ. Values shown represent the averages of 3 experiments. In each experiment, approximately 500 cells were analyzed for each condition.

For single-cell migration assays, cells were seeded sparsely on 3 µg/ml Col-1, and phase-contrast images were captured every 15 min at 37°C and 5% CO_2_ on a Widefield CCD system using a 10× dry lens objective (Carl Zeiss MicroImaging). Migration tracks were generated using ImageJ 1.44, and the average velocity was calculated from approximately 250 cells out of 3 independent experiments.

Proliferation was investigated by seeding cells in 6-well plates, coated with 3 µg/ml Col-1, at a density of 5×10^4^ cells per well, whereafter they were trypsinized and counted every day. Values shown represent the averages of 3 experiments.

### Integrin Internalization and Recycling Assays

Integrin internalization and recycling was investigated essentially as described earlier with some modifications [51]. Briefly, cells on glass coverslips were incubated for 2 hrs at 37°C in serum-free medium, after which they were washed twice in the same medium at 4°C. Cell-surface β1 was then labeled with DyLight 649-conjugated K-20 (10 µg/ml) for 1 hr at 4°C. Immediately after labeling, some coverslips were fixed, and the rest was transferred to 37°C to undergo endocytosis. After 2 hrs, some coverslips were fixed, and the rest was stimulated with 20% FCS for 7.5 min to stimulate recycling of internalized integrins. The cells were fixed, permeabilized with 0.5% Triton and 0.01% saponin, and then processed for confocal microscopy as described above. The number of cells with recycled integrins was scored from confocal images (∼120 cells out of 3 independent experiments).

## Supporting Information

Figure S1 **Ln-332 deposition is not impaired in KS cells.** Ln-332 deposition (green) in NHK and KS cells. Keratinocytes derived from a Junctional Epidermolysis Bullosa (JEB) patient, carying a mutation in the *LAMC2* gene encoding the γ2 chain of Ln-332, were included as a negative control. F-actin, red. Bar, 20 µm.
(TIF)

Figure S2 **Integrin expression in NHK and KS cells.** Cell-surface expression of α3, α2, α5, and β4 subunits on NHK and KS cells was measured by flow cytometry.
(TIF)

Figure S3 **Overexpression of kindlin-1 in NHK cells promotes β1 cell-surface expression and cell spreading.** **A**) Western blot showing overexpression of eGFP-kindlin-1 in NHK cells (NHK^kind-1^). **B**) FACS histogram showing β1 cell-surface expression on NHK and NHK^kind-1^ cells. **C**) Phase/contrast images of NHK and NHK^kind-1^ cells on Col-1 (top) and quantification of average cell area (bottom). AU, arbitrary units. Bar, 10 µm.
(TIF)

Movie S1 **Dynamics of mCherry-vinculin in KS cells.** Dynamics of mCherry-vinculin at the cell-substratum interface were monitored by TIRF microscopy on Col-1-coated glass coverslips. Penetration depth 90 nm, image interval 30 sec, total time 30 min. ImageJ lookup table ‘fire’ was used to enhance visibility.
(AVI)

Movie S2 **Dynamics of mCherry-vinculin and eGFP-kindlin-1 in KSK cells.** Dynamics of mCherry-vinculin (left) and eGFP-kindlin-1 (right) at the cell-substratum interface were monitored by TIRF microscopy on Col-1-coated glass coverslips. Penetration depth 90 nm, image interval 30 sec, total time 30 min. ImageJ lookup table ‘fire’ was used to enhance visibility.
(AVI)

Movie S3 **Dynamics of mCherry-vinculin and eGFP-kindlin-1^del581^ in KSK^del581^ cells.** Dynamics of mCherry-vinculin (left) and eGFP-kindlin-1 (right) at the cell-substratum interface were monitored by TIRF microscopy on Col-1-coated glass coverslips. Penetration depth 90 nm, image interval 30 sec, total time 30 min. ImageJ lookup table ‘fire’ was used to enhance visibility.
(AVI)
