# Supplementary figures and images for: Kindlin-1 Regulates Integrin Dynamics and Adhesion Turnover
Source: PLoS One. 2013 Jun 11;8(6):e65341. doi: 10.1371/journal.pone.0065341 (PMC3679067; doi:10.1371/journal.pone.0065341)

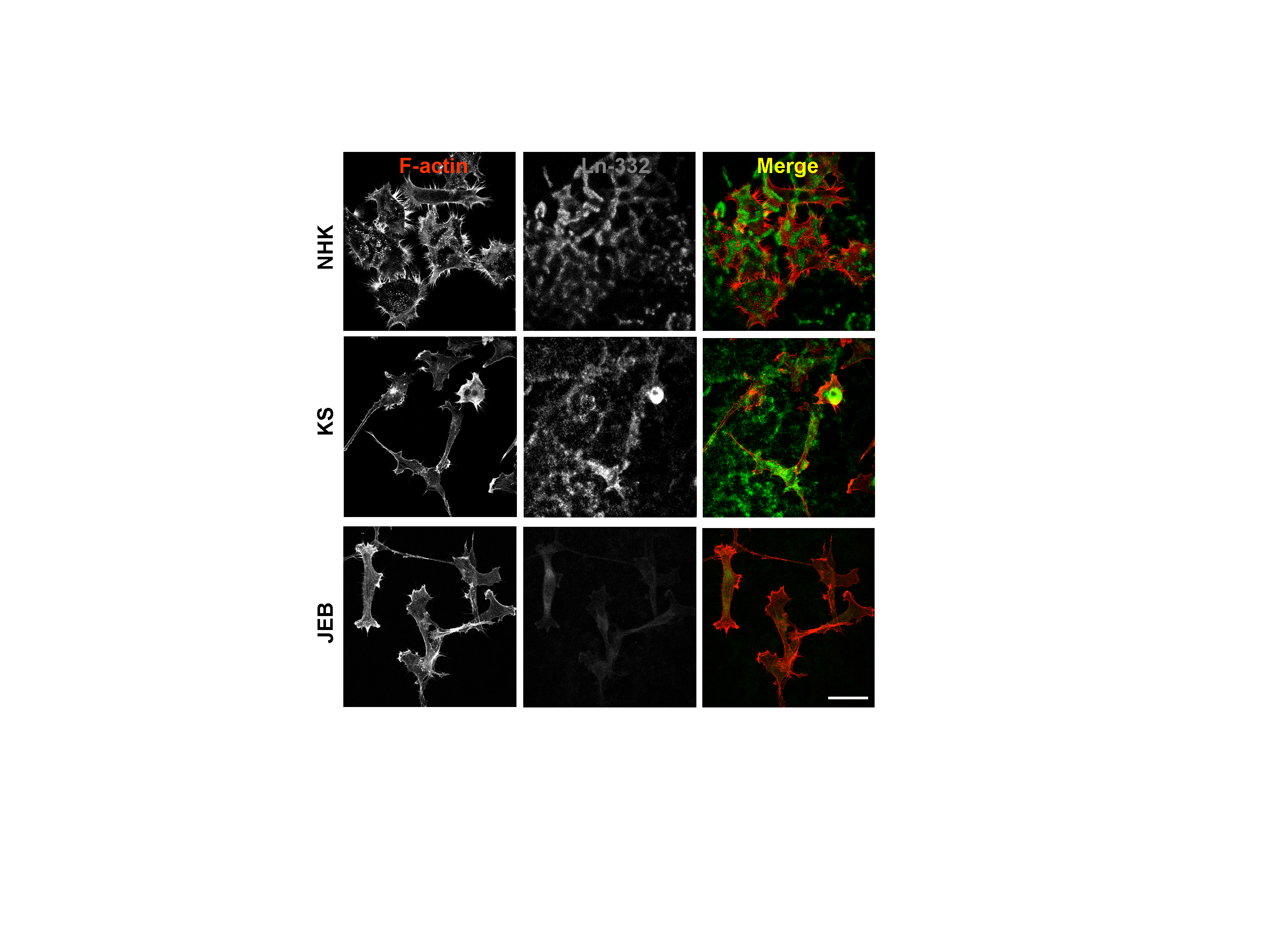

Supplement: Figure S1 — Ln-332 deposition is not impaired in KS cells. Ln-332 deposition (green) in NHK and KS cells. Keratinocytes derived from a Junctional Epidermolysis Bullosa (JEB) patient, carying a mutation in the LAMC2 gene encoding the γ2 chain of Ln-332, were included as a negative control. F-actin, red. Bar, 20 µm. (TIF) [file pone.0065341.s001.tif]

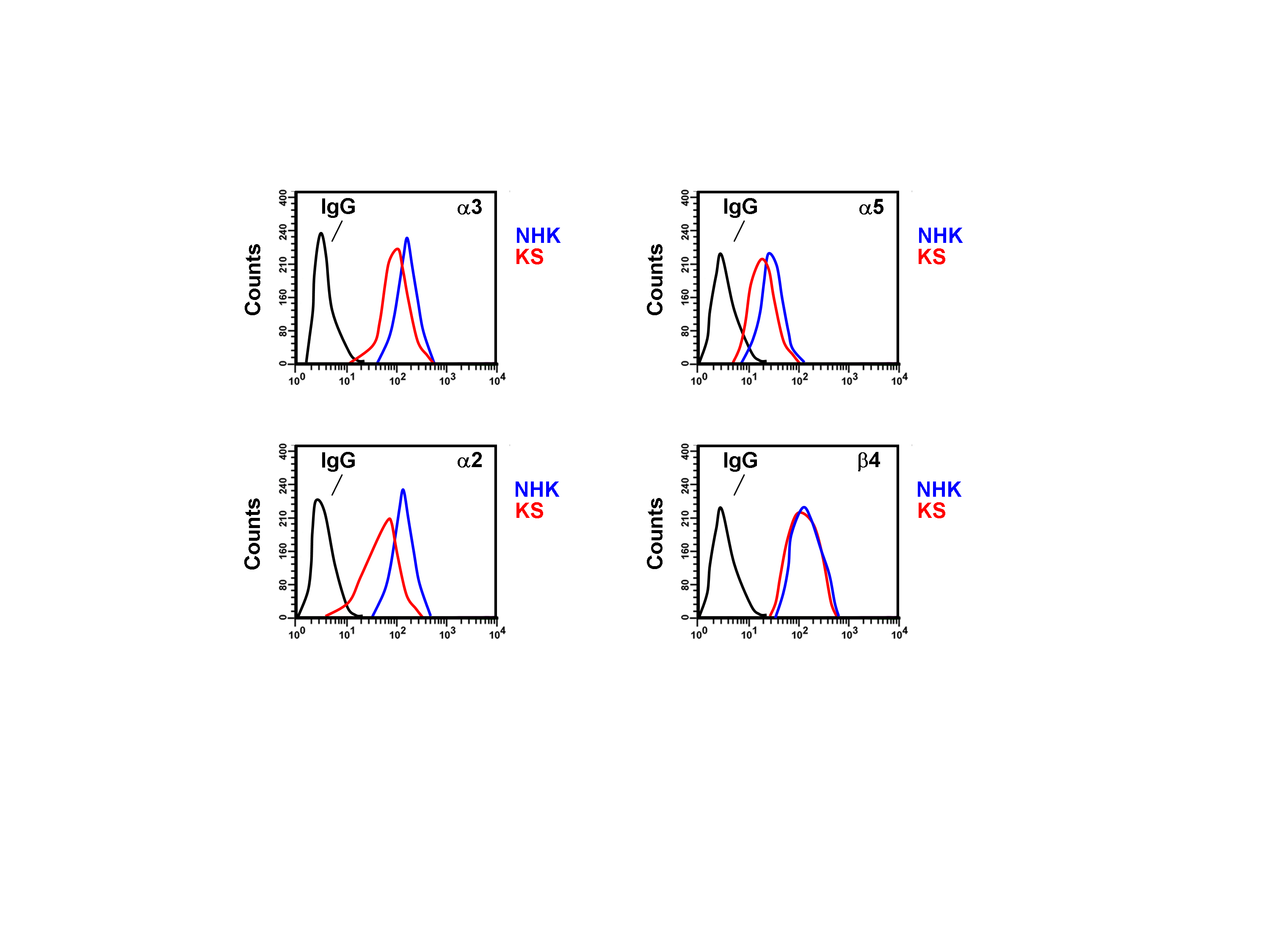

Supplement: Figure S2 — Integrin expression in NHK and KS cells. Cell-surface expression of α3, α2, α5, and β4 subunits on NHK and KS cells was measured by flow cytometry. (TIF) [file pone.0065341.s002.tif]

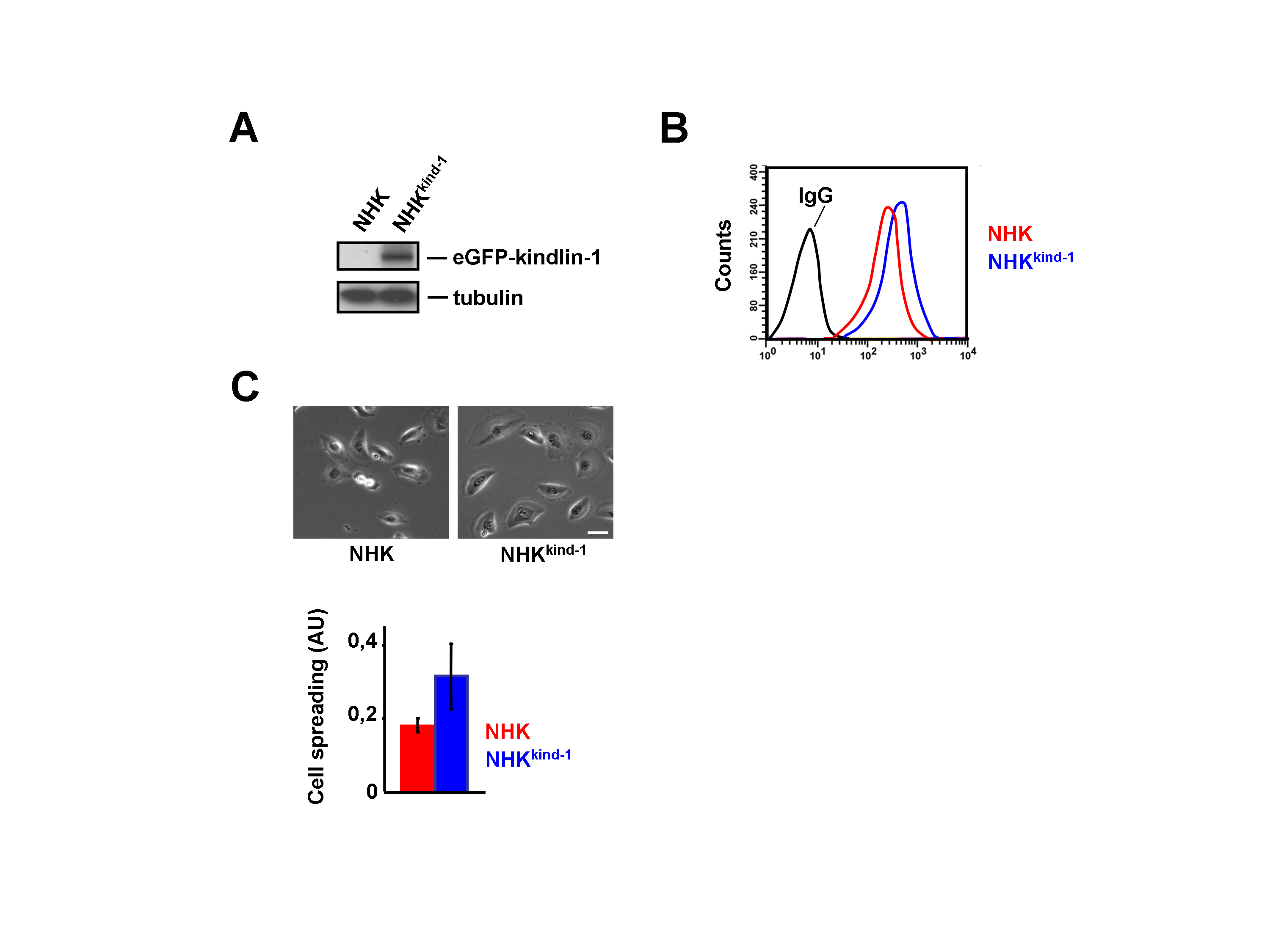

Supplement: Figure S3 — Overexpression of kindlin-1 in NHK cells promotes β1 cell-surface expression and cell spreading. A) Western blot showing overexpression of eGFP-kindlin-1 in NHK cells (NHKkind-1). B) FACS histogram showing β1 cell-surface expression on NHK and NHKkind-1 cells. C) Phase/contrast images of NHK and NHKkind-1 cells on Col-1 (top) and quantification of average cell area (bottom). AU, arbitrary units. Bar, 10 µm. (TIF) [file pone.0065341.s003.tif]
